# Objective structured clinical examination as a competency assessment tool of students’ readiness for advanced pharmacy practice experiences in South Korea: a pilot study

**DOI:** 10.1186/s12909-023-04226-z

**Published:** 2023-04-12

**Authors:** Yun-Kyoung Song, Eun Kyoung Chung, Young Sook Lee, Jeong-Hyun Yoon, Hyunah Kim

**Affiliations:** 1 College of Pharmacy, Daegu Catholic University, 13-13 Hayang-Ro, Hayang-Eup, Gyeongsan-Si, Gyeongbuk 38430 Republic of Korea; 2 College of Pharmacy, Kyung Hee University, 26 Kyungheedae-Ro, Dongdaemun-Gu, Seoul, 02447 Republic of Korea; 3 College of Pharmacy, Keimyung University, 1095 Dalgubeol-Daero, Dalseo-Gu, Daegu, 42601 Republic of Korea; 4 College of Pharmacy, Pusan National University, 2 Busandaehak-Ro 63beon-gil, Geumjeong-Gu, Busan, 46241 Republic of Korea; 5 College of Pharmacy, Sookmyung Women’s University, 100 Cheongpa-Ro 47-Gil, Yongsan-Gu, Seoul, 04310 Republic of Korea

**Keywords:** Objective structured clinical examination, Competency assessment, Pharmacy students, Introductory pharmacy practice experience, Advanced pharmacy practice experience

## Abstract

**Background:**

The assessment of pharmacy students’ readiness to begin the education of an advanced pharmacy practice experience (APPE) in clinical pharmacy settings continues to gain increasing attention. This study aimed to develop an objective structured clinical examination (OSCE) in the core domains acquired through an introductory pharmacy practice experience (IPPE), for evaluating its appropriateness as a tool of assessing clinical pharmacist competency for APPEs in Korean pharmacy students throughout a pilot study.

**Methods:**

OSCE’s core competency domains and case scenarios were developed through a literature review, ideation by researchers, and external experts’ consensus by a Delphi method. A prospective single-arm pilot test was conducted to implement the OSCE for Korean pharmacy students who completed a 60-h course of in-class simulation IPPE. Their competencies were assessed by four assessors in each OSCE station with a pass-fail grading system accompanied by a scoring rubric.

**Results:**

OSCE competency areas including patient counseling, provision of drug information, over-the-counter (OTC) counseling, and pharmaceutical care services were developed with four interactive and one non-interactive cases. Twenty pharmacy students participated in the OSCE pilot test, and their competencies were evaluated by 20 assessors. The performance rate was the lowest in the area of patient counseling for a respiratory inhaler (32.1%) and the highest (79.7%) in OTC counseling for constipation. The students had an average performance rate of 60.4% in their communication skills. Most participants agreed on the appropriateness, necessity, and effectiveness of the OSCE in evaluating pharmacy students’ clinical performance and communication skills.

**Conclusions:**

The OSCE model can be used to assess pharmacy students’ readiness for off-campus clinical pharmacy practice experience. Our pilot study suggests the necessity of conducting an OSCE domain-based adjustment of difficulty levels, and strengthening simulation-based IPPE education.

## Introduction

The pharmacy educational system in South Korea was reformed to a six-year (2 + 4) program in 2009. The major change comprised introducing pharmacy practice experiences to cultivate students’ competencies by enabling them to acquire knowledge, skills, and attitude to perform a pharmacist’s role in various practical fields [1]. The experiential education program consists of two phases: the introductory pharmacy practice experience (IPPE) courses for 60 h, where students are exposed to simulated pharmacy practice environments within the college of pharmacy, and the advanced pharmacy practice experience (APPE) courses of training in hospital and community pharmacy track, industrial/administrative pharmacy track, or pharmacy research track for 1340 h [2, 3].

The assessment of pharmacy students’ readiness to begin APPE education in clinical pharmacy settings continues to gain increasing attention [4, 5]. The Accreditation Council for Pharmacy Education (ACPE) in the United States (US) has emphasized the importance of competency assessment with comprehensive, formative, and summative testing [6, 7]. Since pharmacy practice training was first implemented eighth years ago in South Korea, preceptors and students have raised concerns related to experiential education, such as differences in IPPE educational content and quality among 37 colleges of pharmacy and differences in students’ competence in translating knowledge levels into practice [8, 9]. Despite the apparent need for a competency assessment program to assess students’ readiness for experiential learning, there are no established standardized examinations or evaluation criteria to assess students’ clinical performance consistently and accurately.

Objective structured clinical examination (OSCE) was first introduced as a novel method of assessing the clinical competence of medical students and has been adapted to numerous other health professional programs, including doctor of pharmacy curricula. The OSCE is advantageous in evaluating competency in difficult-to-assess areas, such as communication, problem solving, and decision-making, with relatively high reliability, validity, and objectivity [10, 11]. In the US and Canada, the OSCE has been implemented in doctor of pharmacy programs or national pharmacist licensure examinations to evaluate whether pharmacy students have the necessary knowledge, skills, and attitudes for clinical practice [12, 13]. Standardized OSCE models have been developed in countries including the US, the United Kingdom (UK), Canada, Japan, Malaysia, and the Middle East, for examining the progress in assessing students’ readiness for clinical practice [5, 14–20]. However, there is no generalized assessment program to determine the performance readiness of pharmacy students prior to beginning APPEs. The OSCEs have been developed in the US for assessing competencies acquired during an IPPE [5, 15]. Nevertheless, it is difficult to apply it for the outcome assessment of education only with in-class simulation system, since the IPPEs in the US includes the off-campus training [2, 6, 7]. This study aimed to develop an OSCE in the core domains acquired through an IPPE, to evaluate its appropriateness as a tool for assessing clinical pharmacist competency for APPEs in Korean pharmacy students, throughout a pilot study.

## Methods

### OSCE development

**Fig. 1:**
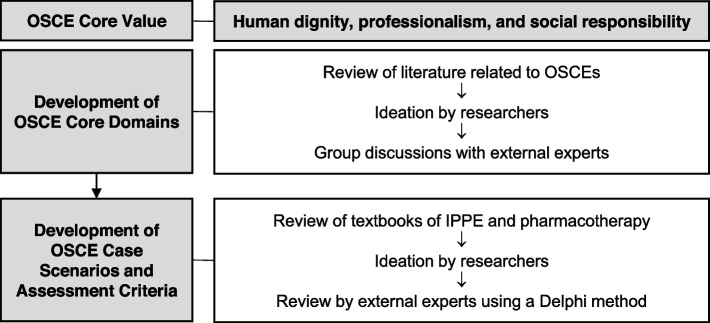
A flowchart for OSCE development

To establish the OSCE blueprint, we set its core values as human dignity, professionalism, and social responsibility (Fig. 1) [6–8]. The OSCE’s core competency domains demonstrated by pharmacy students who completed the IPPE courses, were selected through review of literature related to OSCEs for pharmacy students and pharmacists’ practice examination in the US, the UK, Canada, and Japan [3–8, 12–24], ideation by researchers, and group discussions with experts. We primarily referenced duties for clinical performance test, suggested by Han et al., and the Korean official textbook of IPPE [22, 25].

To develop OSCE cases related to each OSCE competency domain, we identified case objectives and explored possible case scenarios related to each OSCE topic based on the textbooks of IPPE and pharmacotherapy used in 37 colleges of pharmacy in Korea [25, 26] and ideation by the researchers. Subsequently, we finalized the simulated case scenarios and assessment criteria for the clinical performance and communication skills of the students within the given time constraints (i.e., 10 min for each case) through review by external experts qualified for the education of clinical pharmacy and pharmacy practice. They reviewed the OSCE cases and competency criteria to achieve a consensus by the Delphi method [27]. The case scenarios consisted of the title, interactive/non-interactive, purpose of the OSCE, time, materials, instructions for students and questions, instructions for standardized patients/physicians, and instructions for assessors (i.e., answer and assessment criteria). The instructions for standardized patients/physicians contained a specific script with an information guide on the reactions of standardized actors to students’ responses. Development of an assessment criteria along with a scoring rubric, helped evaluate clinical performance skills of pharmacy students, such as critical thinking, patient-centered problem solving, overall attitude and behavior, and provision of correct information, as well as their communication skills, according to each OSCE topic [6–8, 25, 28].

### Setting and subjects

A pilot study was designed as a prospective single-arm observational trial to evaluate the clinical competency of pharmacy students in South Korea prior to APPEs by implementing the OSCE models developed in this study. The inclusion criterion for participants was third-year students of pharmacy schools (i.e., fifth-grade at South Korean pharmacy colleges), who had completed 60 h of IPPE. They were recruited via flyers posted on the website of the Korean Association of Pharmacy Education and four colleges of pharmacy located in Daegu and Gyeongsangbuk-do, South Korea. Assessors who were preceptors of APPEs or clinical faculty members at pharmacy colleges with at least two years of IPPE or APPE educational experience were asked to participate in the study by the researchers to assess the clinical pharmacist competency of the students. On the day for the pilot test, all students or assessors attended their own respective briefing session. They were informed about the OSCE procedure, the total expected time for the exam or assessment, number of simulated cases, and stations with standardized patients/physicians, as well as the survey after the OSCE’s completion. The competency evaluation criteria and rubric scoring methods were explained to the assessors. All participants submitted a written spontaneous participation consent before enrolling in the study.

This pilot study was conducted at Keimyung University’s College of Pharmacy in South Korea, on September 26, 2020. Each core domain of the OSCE was examined at five stations, using a mock pharmacy and standardized patients/physicians with simulated tasks or problems. Prior to the day of the OSCE, four actors serving as patients or physicians received a 30-min training from the primary investigators, along with scripts for the simulation. On the day for the pilot test, students were randomly assigned to four groups of five, and took the exam at five stations for each OSCE case. Each case required 10 min, that is, two minutes of preparation time in front of the station, seven minutes of test time, and one minute of travel time to the next station. A research assistant in each station managed the time schedule, and two coordinators facilitated the overall OSCE process. A small amount of allowance for research participation was paid to all participants (i.e., students or assessors), standardized patients/physicians, coordinators, and assistants.

### Assessors and scoring

The assessors were provided with competency assessment mark sheets with a binary grading system (i.e., pass or fail) developed according to each OSCE case in this study. Four assessors assigned to each station based on the peer investigator's judgement, evaluated the competencies of all students as per the provided criteria. The pass-fail grading system is appropriate to assess the clinical simulation performance of the healthcare students [29, 30]. The assessors were provided with specific exemplar answers for each evaluation criterion to make their assessment similar. For each evaluation criterion, the students received either a pass or a fail on their clinical performance or communication skills, from the assessors with the following standards: (1) a pass when meeting 50% or more of each assessment criterion for clinical performance and communication skills and (2) a fail when meeting less than 50% of each assessment criterion. Consequently, the students and assessors conducted surveys with a four-point Likert scale on the difficulty, usefulness, and satisfaction of each OSCE competency area. Students were informed that they could individually attain their assessment results if desired.

### Analysis

Data are shown as numbers and percentages for categorical variables, and as means and standard deviations (SD) for continuous data. The difficulty of the OSCEs was evaluated based on the students’ performance rates which were measured by the percentage of students who passed each assessment criterion. The OSCE was considered difficult if the performance rate was less than 40% and easy if the rate was greater than 80% [31, 32]. Fisher’s exact and chi-square tests were used to compare the clinical performance assessment results between professors and hospital/community pharmacists as well as survey results between students and assessors. Statistical significance was set at a two-sided *p*-value of < 0.05, and data analysis and computation were conducted using SPSS Statistics (version 22.0; IBM, Armonk, NY, USA).

## Results

### Development of OSCE’s core competency domains and cases

We finally determined five core competency domains for the assessment of pharmacy students’ readiness for the APPEs, which were: (1) patient counseling; (2) prescription review; (3) provision of drug information; (4) over-the-counter (OTC) counseling; and (5) pharmaceutical care service. The prescription review area was selected instead of the drug preparation and dispensing areas suggested by Han et al. [22], since researchers determined that the competencies related to dispensing should be evaluated after the APPEs [25]. The detailed topics and general assessment criteria related to each OSCE’s core competency domain are listed in Table 1. Of the five competency domains, four were interactive examinations involving standardized patients/physicians, and only the prescription review area was a non-interactive one, where the student had to solve problems and present them to the assessors. Summaries of the simulated case scenarios for the five competency areas are presented in Table 2. For example, the objectives of the patient counseling case were to counsel and educate a patient with asthma on a dry-powder inhaler. This station simulated a 25-year-old man who came to the pharmacy with a prescription for a fluticasone inhaler, and the student was asked to provide appropriate patient education and counseling. A four-level rubric was set to score students’ competency in the OSCE, including (1) outstanding for the achievement of 90% or more, (2) clear pass for 70 − 89%, (3) borderline pass for 50 − 69%, and (4) clear failure for less than 50%.

**Table 1:** Topics and assessment criteria of the OSCE core competency domains

Core competency domain	Type	Detailed topics	General assessment criteria related to clinical pharmacist competency
Patient counseling	Interactive	• Counseling and providing information for an inhaler	• To counsel a patient for the prescription drugs effectively and correctly • To provide key-points needed for a patient’s understanding appropriately • To confirm the patient’s understanding of the delivered information • To use language understandable to a patient • To communicate with a patient effectively
Prescription review	Non-interactive	• Medication use review of a prescription for a pediatric patient	• To review the appropriateness of the prescription based on the legal standard and patient’s clinical conditions • To evaluate drug-related problems of the prescription and suggest appropriate solutions • To dispense a prescription and check its accuracy
Drug information service	Interactive	• Drug identification • Providing drug-related information to health care professionals	• To identify the partner’s requirements through conversations • To provide appropriate information on inquiries of a partner (i.e., healthcare professional or patient) based on the adequate evidence • To use language understandable to a partner • To communicate with a partner effectively
OTC counseling	Interactive	• Selection of OTC products for diarrhea	• To identify the symptoms and requirements through conversations with a patient • To recommend appropriate OTC medicines to a patient • To suggest appropriate non-pharmacotherapy information • To use language understandable to a patient •To communicate with a patient effectively
Pharmaceutical care service	Interactive	• Corticosteroid dose equivalents	• To perform calculations for dosage adjustment according to changes of the formulation or drugs • To adjust dosage or administration methods according to patient clinical/demographic condition or results of therapeutic drug monitoring • To use language understandable to a patient • To communicate with a patient effectively

**Table 2:** Summaries of the cases in each OSCE core competency domain

	**OSCE core competency domains**				
	**Patient counseling**	**Prescription review**	**Drug information service**	**OTC counseling**	**Pharmaceutical care service**
Title	Counseling and providing information for an inhaler	Medication use review	Drug identification and providing drug information to health care professionals	Selection of OTC products for diarrhea	Corticosteroid dose equivalents
Format	Interactive station	Non-interactive station	Interactive station	Interactive station	Interactive station
Objectives	To assess a student’s ability to counsel appropriately for a patient who are using an inhaler	To assess a student’s ability to review prescriptions, identify prescription errors, and solve clinical management problems	To assess a student’s ability to identify the drug information needs of the requestor and to describe the application in formulating responses and recommendations	To assess a student’s ability to identify patient’s symptoms and recommend appropriate OTC medication to help patients deal with their health issues	To assess a student’s ability to evaluate, design, and recommend optimal pharmacotherapy for the management of specific patients
Time allotted for the task	Total 10 min—preparation for encounter, 2 min; encounter, 7 min; time between groups, 1 min				
Station requirements	Sample prescription and inhaler	Sample prescription and package insert	Sample medicine (Paxil CR tablet 12.5 mg ®), computers, internet, drug information databases and pharmacotherapy textbooks	Nonprescription drugs (OTC) textbook and package inserts	Calculator, corticosteroid dose equivalents table, package inserts of intravenous methylprednisolone and oral prednisolone, and pharmacotherapy textbooks
Directions to the student	A 25-year-old male comes to the pharmacy with a prescription for fluticasone inhaler. Provide appropriate information and counseling	You are a pharmacist working in a community pharmacy. After reviewing the prescription for the pediatric patient, answer the following questions Q1. Evaluate the legal appropriateness of a prescription. Does the prescription contain all of the information? Q2. Evaluate the appropriate dose for a prescription Q3. What action(s) would you recommend? Explain your answer	You are a clinical pharmacist working at drug information center in a hospital. You are approached by Dr. Lee and he requests a drug identification	A 25-year-old female comes to the pharmacy for diarrhea. Identify the patient’s symptoms and recommend appropriate OTC medication. Provide patient education and counseling	You are a clinical pharmacist working at a teaching hospital who specializes in internal medicine. A physician on your team asks steroid dosing for a patient with ulcerative colitis
Scenario information	• Name: Mr. Kim • Patient age: 25 years • Past medical history: none • Social history: no smoking, no drinking	NA	• Name: Dr. Lee, MD • Age: NA • Appearance and affect: dressed in white coat, cooperative	• Name: Mr. Park • Patient age: 30 years • Chief complaints: I have diarrhea with abdominal pain. This hasn’t stopped since yesterday and I didn’t eat anything. I had seafood pasta for lunch yesterday. I need to go to work and wish to stop this as quickly as possible • Past medical history: none • Current medication: occasionally pain medication for migraine • Social history: no smoking, no drinking	• Name: Dr. Kim, MD • Age: NA • Appearance and affect: dressed in white coat, cooperative
Directions to the standardized participant	You came to the pharmacy to be counseled on how to use an inhaler for your asthma. If the student asks if you have any experience in using an inhaler before, answer that you are currently using a salbutamol inhaler. After the student's counseling on how to use the inhaler, you can ask any questions you might have. If the student asks you to demonstrate the use of the inhaler, use it without turning the inhaler lever (wrong usage). If the information is not provided by the student, you will ask the following questions Q1. How many times should I use it? Q2. How do I know if I used the inhaler appropriately? Q3. Do I need to rinse or gargle my mouth with water after using an inhaler?	NA	You are Dr. Lee, an internal medicine resident. Your patient is a 70-year-old female who is taking the following medication. You will ask the following two questions to the student Q1. A 70-year old female patient is taking the following medication. What is the generic or trade name of the medication? Q2. This patient is taking naproxen for osteoarthritis. Can this medication be taken with naproxen?	You came to the pharmacy for diarrhea medication. If the student asks what you need, reply with medication for your diarrhea. After the student recommends the OTC medication, you will ask the following questions Q1. How can I take this medication? Q2. Can I eat something while taking this medication? Q3. I saw other OTC medication on TV advertisement. What are the differences between this product and the one you recommended?	You are Dr. Kim, an internal medicine resident. Your patient is a 42-year-old male with active ulcerative colitis. This patient is taking intravenous methylprednisolone 40 mg. You will ask the following two questions to the student Q1. We are planning to change the intravenous methylprednisolone to oral prednisolone. What is the equivalent dosing for oral prednisolone? Q2. What is the appropriate strategy for prednisolone tapering?

### Implementation of the OSCE in a pilot test

Twenty students from two pharmacy colleges located in Daegu and Gyeongsangbuk-do were included. As shown in Table 3, there were also 20 evaluators from community and hospital pharmacies and colleges of pharmacy with at least two years of educational experience, either in the IPPEs or APPEs.

**Table 3:** Demographic characteristics of the competency assessors of pharmacy students (*n* = 20)

Characteristics	Number of assessors (%)
Age, mean (SD)	45.2 (7.5)
Gender, female	17 (85)
Working areas	
Hospital pharmacist	5 (25)
Community pharmacist	5 (25)
Professors	10 (50)
Academic degree	
BS	2 (10)
MS	4 (20)
PhD	14 (70)
Pharmacy practice education experience	
IPPEs	10 (50)
APPEs	11 (55)
Region	
Metropolitan area	2 (10)
Non-metropolitan area	18 (90)

Table 4 demonstrated that the overall performance rate of the students was 50.8%. The average performance rates were 32.1, 64.8, 65.4, 79.7, and 62.5% in patient counseling, prescription review, provision of drug information, OTC counseling, and pharmaceutical care service, respectively. Among the 18 assessment criteria in patient counseling, the students had a performance rate of less than 40% for the 11 criteria, and no one met the following two criteria: “Explain expected length of the patient counseling session” and “Allow patients to summarize and organize relevant education and counseling.” Among the 14 criteria in the prescription review area, the students’ performance was less than 40% for the following three criteria: “Note that the physician’s signature on the prescription is missing”; “Note that the prescription expiration date is missing”; and “Check the patient’s age.” In drug information provision, students’ performance was relatively low only for two of the six assessment criteria: “Reconfirm the question clearly” and “Provide proper information on naproxen co-administration.” The students’ performance was more than 50% for all four criteria in the OTC counseling. In pharmaceutical care service, students’ performance was low only for the assessment criterion, “The converted daily dose of oral prednisolone was designed using the appropriate number of tablets and frequency, considering the formulation and dose of those on the market.” among the total four criteria. The evaluation results of professors and hospital/community pharmacists differed statistically significantly in five of the six criteria in the drug information area, while only two of the 14 criteria were different in the prescription review area.

**Table 4:** Assessment criteria for clinical performance skills in each OSCE area and assessment results in a pilot study

Core competency domains	Detailed assessment criteria of clinical performance skill	Performance rate of students with successful demonstrations of skill, mean (SD)
Patient counseling	1. Perform a brief greeting and self-introduction	50.0
	2. Identify the patient	10.0
	3. Explain the purpose of patient counseling	31.3 (11.4)^*^
	4. Explain expected length of the patient counseling session	0
	5. Explain the dosage and proper use of inhaled corticosteroids, according to the prescription	47.5 (21.9)^*^
	6. Explain, both verbally and clearly, how to use the inhaler	
	6–1. Hold the body horizontally with your left hand, put your right hand in the handle groove, and turn until you hear a snap	53.8 (24.8)
	6–2. Turn the operating lever until it makes a “clap” sound	50.0 (18.4)
	6–3. Turn your head to the side and exhale all the way to prepare for inhalation	33.8 (18.2)^*^
	6–4. Hold the inlet in your mouth and inhale it all the way, strong and deep	38.8 (20.7)^*^
	6–5. Take the inhaler out of your mouth and hold your breath for approximately 5–10 s to ensure that the drug is absorbed as much as possible	42.5 (21.9)^*^
	6–6. Exhale naturally and slowly through your nose	15.0 (12.7)
	6–7. Hold the handle and turn the lid to the left until you hear a snap	37.5 (13.0)^*^
	6–8. Rinse your mouth and throat with water after inhalation	50.0 (20.3)^*^
	7. Demonstrate the correct use of the inhaler	23.8 (12.4)^*^
	8. Let the patient demonstrate the use of the inhaler	23.8 (5.4)
	9. Watch the patient’s demonstration and provide appropriate feedback	30.0 (12.2)
	10. Ask the patient if they have any questions or concerns	57.5 (5.6)
	11. Allow patients to summarize and organize the relevant education and counseling	0
	Sub-total	32.1 (22.9)
Prescription review	1. Note that the physician’s signature on the prescription is missing	20.0
	2. Note that the prescription expiration date is missing	40.0
	3. Check the patient’s age	30.0 (14.6)^*^
	4. Check the patient’s body weight	77.5 (5.6)
	5. Check the appropriate pediatric dosage by referring to the instructions for use	85.0 (6.1)
	6. Note the inappropriate dose in the prescription	80.0 (11.2)
	7. Note the inappropriate duration of treatment	71.3 (11.4)
	8. Suggest the appropriate dosage to the patient	80.0 (5.0)
	9. Suggest the appropriate daily dose to the patient	78.8 (6.5)
	10. Calculate the proper dose (mg) according to the patient’s body weight	68.8 (8.9)
	11. Convert the calculated dose (mg) to volumes (ml)	73.8 (8.9)
	12. Use the appropriate terms	73.8 (14.3)
	13. Answer with confidence	50.0 (21.2)^*^
	14. Answer with respect to the other person	95.0 (5.0)
	Sub-total	64.8 (23.6)
Drug information service	1. Reconfirm the question clearly	36.3 (34.0)^*^
	2. Find an appropriate drug information resource for the drug’s identification	90.0 (3.5)
	3. Identify the drug using the drug database	91.3 (4.1)^*^
	4. Check the information about drug interaction with Naproxen by using the appropriate data sources or evidence	73.8 (15.6)^*^
	5. Provide proper information on the drug identification results	77.5 (17.5)^*^
	6. Provide proper information on naproxen coadministration	23.8 (12.9)^*^
	Sub-total	65.4 (31.6)
OTC counseling	1. Identify the patient’s symptoms and needs	90.0 (9.4)^*^
	2. Ask for more information to provide the best treatment choice	68.8 (23.3)^*^
	3. Select at least one product and provide information	95.0 (5.0)
	4. Provide information about a diarrhea-related diet	65.0 (9.4)
	Sub-total	79.7 (18.8)
Pharmaceutical care service	1. Check the current dose of methylprenisonlone	70.0
	2. Check the corticosteroid dose equivalents for the dosing conversion from methylprednisolone to oral prednisolone	56.3 (4.1)
	3. The converted daily dose of oral prednisolone was designed using the appropriate number of tablets and frequency, considering the formulation and dose of those on the market	37.5 (21.4)^*^
	4. Inform the patient that after long-term steroid systemic therapy, treatment should be gradually stopped through a tapering process	86.3 (4.1)
	Sub-total	62.5 (21.1)
**Total**		50.8 (29.8)

^*^Statistically significant difference of the assessment results between professors and hospital/community pharmacists (*p* < 0.05 in chi-square or Fisher’s exact test)

As shown in Table 5, the average performance rate in the seven assessment criteria for the students’ communication skills was 60.4%. However, only three criteria were passed by more than 80% of the students: “Use terms and expressions while considering the partner’s capacity for understanding.”; “Adhere to appropriate speech and an attitude that makes the partner (patient or healthcare professional) feel comfortable.”; and “Explain or respond with respect to the partner (patient or healthcare professional).”.

**Table 5:** Assessment criteria for communication skills in OSCEs and assessment results in a pilot study

Detailed assessment criteria of communication skill	Performance rate of students with successful demonstrations of skill, mean (SD)
1. Perform a brief greeting and self-introduction	49.2 (18.9)
2. Use terms and expressions while considering the partner (patient or healthcare professional)’s capacity for understanding	83.3 (13.1)
3. Adhere to appropriate speech and an attitude that makes the partner feel comfortable	83.3 (13.7)^*^
4. Explain or answer with confidence	70.0 (17.0)^*^
5. Explain or respond with respect to the partner	94.6 (6.6)
6. Check if the partner understood or had any other questions	16.9 (15.4)
7. End the conversation properly	42.8 (29.2)
Total	60.4 (31.6)

^*^Statistically significant difference of the assessment results between professors and hospital/community pharmacists (*p* < 0.05 in chi-square or Fisher’s exact test)

Table 6 shows the results of the students’ and assessors’ surveys after the OSCE was implemented. The students and assessors thought that the time for each session of the OSCE was sufficient, the questions were appropriate for evaluating the clinical performance of the students prior to the APPEs, and standardized OSCEs were necessary for the evaluation of students’ clinical pharmacist competencies acquired in the IPPEs. In addition, all of them agreed that students’ clinical performance would improve if OSCEs were conducted in the future. However, statistically, more students than assessors answered that the examinations for prescription review and OTC counseling were more difficult.

**Table 6:** Ratings of students and assessors for OSCEs in a survey with a four-point Likert scale

**Questionnaires**^a^	**Patient counseling, mean (SD)**			**Prescription review, mean (SD)**			**Drug information service, mean (SD)**			**OTC counseling, mean (SD)**			**Pharmaceutical care service, mean (SD)**		
	**Student**	**Assessor**	*p* value^**^	**Student**	**Assessor**	*p* value^**^	**Student**	**Assessor**	*p* value^**^	**Student**	**Assessor**	*p* value^**^	**Student**	**Assessor**	*p* value^**^
The exam was difficult	2.80 (0.93)	2.50 (0.87)	0.21	2.95 (0.80)	2.00	< 0.01	2.20 (0.68)	2.75 (0.43)	0.31	2.65 (0.79)	2.00	0.05	2.75 (0.89)	2.00	< 0.01
The time given for the exam was sufficient	3.50 (0.50)	3.50 (0.50)	1.00	3.45 (0.50)	3.25 (0.43)	0.46	3.50 (0.50)	3.50 (0.50)	1.00	3.50 (0.50)	3.75 (0.43)	0.36	3.50 (0.50)	3.50 (0.50)	1.00
The questions were appropriate for evaluating the clinical performance	3.16 (0.87)	3.25 (0.43)	0.85	3.47 (0.50)	3.00	0.08	3.37 (0.48)	3.00	0.15	3.11 (0.72)	3.50 (0.50)	0.78	3.32 (0.57)	3.25 (0.43)	0.78
Pharmacy OSCEs prior to APPEs are needed in the future	3.65 (0.48)	3.75 (0.43)	0.70	3.50 (0.50)	3.00	0.06	3.60 (0.49)	3.50 (0.50)	0.71	3.65 (0.48)	3.50 (0.50)	0.57	3.50 (0.59)	3.50 (0.50)	1.00
Students’ clinical performance would be improved if the OSCEs were conducted in the future	3.75 (0.43)	3.75 (0.43)	1.00	3.65 (0.48)	3.00	0.02	3.65 (0.48)	3.50 (0.50)	0.57	3.75 (0.43)	3.75 (0.43)	1.00	3.70 (0.46)	3.75 (0.43)	0.84

^**^Statistically significant difference between students and assessors, *p* < 0.05 in chi-square or Fisher’s exact test

^a^Scoring on a four-point Likert scale was as follows: 4-point = strongly agree; 3-point = agree; 2-point = disagree; 1-point = strongly disagree

## Discussion

As there is a need to improve healthcare services in Korea, pharmacy colleges have tried to develop methods to evaluate students’ practical skills and performance [8, 22]. This pilot study showed that application of the OSCE to Korean students completed in-class pharmacy simulation courses was feasible to assess their competencies and preparedness for the advanced training in community or hospital pharmacies. To the best of our knowledge, this is the first study to apply a standardized OSCE system to pharmacy students who completed 60-h IPPE courses at Korean colleges of pharmacy, in order to evaluate their clinical performance for the APPE curriculum comprehensively and objectively.

This OSCE model can evaluate nine of the 11 pre-APPE core domain competencies of the ACPE: patient safety; basic patient assessment; medication information; identification and assessment of drug related problems; mathematics applied to dose calculation; professional and legal behavior; general communication abilities; counseling patients; and drug information analysis and literature research [6, 7]. Unlike the Korean IPPE curriculum, the one in the US mainly includes off-campus practice in community and hospital pharmacies as along with in-class simulation training [6, 7]. The Pharmaceutical Common Achievement Tests Organization in Japan presented five OSCE competency areas, including patient counseling, dispensing, dispensing audit, aseptic dispensing, and provision of drug information [17]. The OSCE core domains in this study areas were developed after considering the IPPE curriculum and its official textbook, utilized by most Korean pharmacy schools [3, 25]. Counselling patients for complex dosage forms (i.e., respiratory inhalers or self-injection devices) or OTCs, has been a key competency for clinical pharmacists to provide effective health and medication information to patients, and confirm their understanding of it [6, 7, 25, 33]. By assessing students’ ability towards patient care and prescription review, we could evaluate their basic knowledge, critical thinking, and problem-solving competencies, for assessing patient conditions and DRPs in the community or hospital pharmacy. The competency of time bound drug information analysis and literature research, could be assessed by the area of drug information service, which required the use of adequate drug information resources and evidence-based pharmacotherapy, to provide safe and effective pharmacotherapy [6, 7, 15, 25, 26].

The OSCE stations with standardized patients or physicians were appropriate since pharmacy students have been recommended to complete a specific clinical task often in an interactive environment [21, 33, 34]. The students’ average performance was the lowest at 32.1% in the case of counseling the patient with the inhaler, and the highest at 79.7% in the OTC counseling. This might indicate towards the insufficient readiness of students, for counseling patients with prescribed inhalers at the community or hospital pharmacies. Contradictorily, the students found the cases related to prescription review and pharmaceutical care service, as well as patient counseling, difficult. In Korean pharmacy schools, the IPPE curriculum is operated as in-class simulation of prescription review, dispensing, medication therapy management, patient counseling, and drug information provision, while the APPE courses are conducted as field training at community or hospital pharmacies [2, 3]. Since participating students had not yet started APPE courses, the OSCE cases proved difficult, which resulted in their performance rate dropping below 80%, in certain criteria of all OSCE areas. Malaysian pharmacy students also considered the OSCE areas related to patient counseling, drug dosage review, and drug information service relatively difficult, compared to the areas related to drug-related problems or pharmacokinetics [19]. Despite pharmacists being required to counsel patients within the expected duration, and verify patients’ medication knowledge according to the pharmacist-conducted patient counseling guidelines and the textbook used in the Korean college of pharmacy, no student met the relevant assessment criteria [25, 34, 35]. This study also showed that students portrayed weaknesses at the beginning and end of the communication in clinical pharmacy practice. Contradictorily, Japanese students showed excellent outcomes in most communication skill areas, which was probably affected by the list of tasks provided a minute before the advanced OSCE [36]. The standardized IPPE curriculum applied to all colleges of pharmacy in South Korea is limited. It was reported that the incorporation of simulation based IPPE made pharmacy students more confident on technical and communication skills, and more aware of medication errors and other patient safety issues [15]. Therefore, Korean pharmacy colleges’ IPPE education should strengthen their curriculum based on simulation education, for applying the knowledge to actual clinical situations related to the five key competency areas, and involve the preceptors as reviewers to reduce the differences in the outcome assessment.

The pharmacy students, faculty, and preceptors, need to be introduced to the OSCE system. Implementation of OSCEs as part of the evaluation of clinical performance could help students improve their capabilities by identifying their current level via the assessment of their performances [14, 37]. In a study with third-year pharmacy students in the US, the OSCE was also found to be a means to evaluate students’ clinical capabilities obtained through IPPE practices [2, 5, 7]. Therefore, additional cases related to each OSCE core domain should be developed with the validated assessment criteria through continuous discussions between the pharmacy colleges’ faculty members and APPEs' preceptors. It is also necessary to standardize the OSCE content and lay the foundations for the OSCE introduction by referring to the OSCE system of other healthcare professionals to integrate the OSCE into the pharmacy curriculum. This might be ensured through the development of guidelines, which include details of the time of OSCE, eligibility, management of students, the execution of the exam (i.e., time, location, duration, etc.), assessment methods, and continuous quality management as well as the development and confidentiality of the OSCE cases/questions, by referring to this pilot study [21, 34]. However, the OSCE's adoption should consider an enormous budget allocation for space, administrative overhead, and faculty members’ time [13]. Further studies are needed to find a cost-effective way of introducing the OSCE in Korean pharmacy educational system.

Although several countries such as the US, Canada, Australia, the United Kingdom and Japan have used the OSCE in various ways for evaluating clinical competencies of pharmacy students, most pharmacy schools around the world have not yet introduced or are preparing to introduce the OSCE in their pharmaceutical education systems [21, 38]. Therefore, other countries or organizations could refer to the OSCE model developed in this study to develop or improve the OSCE system for competency assessment of students’ readiness for the pharmacy practice experiences in community or hospital pharmacies.

As this is the first study to implement an OSCE in Korea, some problems were encountered during the pilot test. First, only one case was developed for each core competency domain and implemented to a limited number of students in this pilot project, which could confound the OSCE’s assessment outcomes. Thus, it is desirable in the future to develop various simulated case scenarios and questions, and implement the OSCE on a larger number of students. Second, the scoring rubric and questionnaire used in this study lacked proper calibration or validation. Considering that the standardized scoring rubrics are essential for improving the assessment's consistency, further studies are needed to calibrate examiners on the use of rating scales before adopting the OSCE for Korean pharmacy students [28]. Finally, most of the pharmacy students hold insignificant information about the OSCE system, although we explained the overall OSCE procedure to the participants, in a briefing session before the pilot study. Therefore, some students might have difficulties in understanding the simulation based OSCE process and test questions. It was reported that the awareness of the simulated situation made students feel slightly unreal, where only 77% of the students speaking to the simulated patients felt like a real doctor [39]. Moreover, performance anxiety in certain students and examination unfamiliarity in both students and evaluators, probably caused relatively low performance rates in certain domains [40].

## Conclusion

The OSCE for patient counseling, prescription review, drug information provision, OTC counseling, and pharmaceutical care services, can be used to assess those pharmacy students’ clinical competencies, who completed the 60-h IPPE course in a pharmacy college. Our pilot study suggests the necessity of an OSCE domain-based difficulty adjustment, and the strengthening of simulation-based IPPE education, through continuous discussion between the pharmacy faculties and preceptors for pharmacy students’ readiness to practice off-campus clinical pharmacy operations. Future studies are needed to validate this observation’s feasibility in large numbers of pharmacy students.

## Data Availability

The full contents of the OSCE cases and dataset generated and analyzed during the pilot study are available from the corresponding author on reasonable request.

## Abbreviations

ACPE: Accreditation council for pharmacy education
APPE: Advanced pharmacy practice experience
BS: Bachelor of science
IPPE: Introductory pharmacy practice experience
MS: Master of science
NA: Not available
OSCE: Objective structured clinical examination
OTC: Over-the-counter
PhD: Doctor of philosophy
SD: Standard deviations
US: The United States
